# Estimation of the Dietary Acrylamide Exposure of the Turkish Population: An Emerging Threat for Human Health

**DOI:** 10.3390/nu16183088

**Published:** 2024-09-13

**Authors:** Burhan Basaran

**Affiliations:** Department of Nutrition and Dietetics, Faculty of Health Sciences, Recep Tayyip Erdogan University, Rize 53100, Türkiye; burhan.basaran@erdogan.edu.tr

**Keywords:** acrylamide, dietary exposure, carcinogenic risk, hazard index, margin of exposure, good scenario, bad scenario

## Abstract

Acrylamide is a contaminant formed during heat treatment that poses potential health risks and occurs naturally in foods. Therefore, it is crucial to evaluate exposure from the consumption of foods containing acrylamide since dietary exposure continues throughout life. In this study, the acrylamide exposure level of people living in Türkiye is estimated. Consumption of a total of 28 foods in 9 different food groups was calculated using a deterministic model under two different scenarios. The exposure levels were evaluated in terms of carcinogenic, non-carcinogenic and neurotoxic health risks. The daily total acrylamide exposure levels of individuals aged 15 and older were determined as 58 µg/day (0.85 µg/kg bw/day) and 196 µg/day (2.80 µg/kg bw/day) for the good and bad scenarios, respectively. The highest daily acrylamide exposure in the good scenario came from brewed black tea (29%), whereas French fries (50%) were the source of highest daily acrylamide exposure in the bad scenario. According to the hazard index (HI) and margin of exposure (MOE) data, the good scenario (all food) is considered safe, while the bad scenario (all food) has potential and serious health risks. According to the carcinogenic risk (CR) data, both scenarios carry significant health risks. It is therefore important that consumers, producers and official institutions collaborate and take measures to reduce acrylamide exposure.

## 1. Introduction

Acrylamide (CAS No: 79-06-1; C_3_H_5_NO) is a colorless, odorless, crystalline chemical compound that is easily soluble in water, methanol and acetone and has a widespread industrial use (dams, tunnels, paper, laboratories, etc.) [1].

Acrylamide is a toxic compound [2] and classified as probably carcinogenic to humans in Group 2A by The International Agency for Research on Cancer [3]. The European Commission categorizes acrylamide as a Category 2 reproductive toxicant, a Category 1B carcinogen and mutagen [4]. The European Chemical Agency has included acrylamide in its List of Substances of Very High Concern [5]. The European Food Safety Authority (EFSA) has announced that acrylamide can react with DNA, RNA and proteins to form compounds with genotoxic and neurotoxic effects [6,7]. Different studies have reported that acrylamide intake may cause various birth defects [8,9,10], damage the peripheral, autonomic and central nervous systems [11,12], and increase the risk of developing various types of cancer in respiratory, reproductive and gastrointestinal systems [13,14,15,16,17].

The presence of acrylamide in foods was first demonstrated in 2002 [18]. Although there are some uncertainties regarding the formation of acrylamide in foods, the Maillard reaction is accepted as the main mechanism [19]. In addition, other formation mechanisms called the acrolein pathway have been identified in foods [20]. The level of acrylamide in foods varies according to many factors such as the type of raw material, chemical composition of the raw material, climatic conditions, production and storage conditions, and temperature and time applied during the preparation of foods [6]. The European Commission has established benchmark levels for acrylamide in certain foods: French fries (ready-to-eat) 500 µg/kg; potato-based crisps, crackers, and other potato products 750 µg/kg; roast coffee 400 µg/kg; instant (soluble) coffee 850 µg/kg; coffee substitutes 500–4000 µg/kg; biscuits and wafers 350 µg/kg; crackers (except potato-based) 400 µg/kg; crispbread 350 µg/kg; gingerbread 800 µg/kg; soft bread 50–100 µg/kg; breakfast cereals 150–300 µg/kg; baby foods (processed cereal-based foods) 40 µg/kg; and biscuits and rusks (for infants and young children) 150 µg/kg. The European Commission has recommended monitoring acrylamide content in food since 2007 [21].

Acrylamide research continues to maintain its importance since acrylamide is found at different levels in many foods frequently consumed in daily life. Acrylamide exposure from the consumption of these foods continues throughout life with potential health risks. The European Commission and the Joint FAO/WHO Expert Committee to Food Additives (JECFA) have emphasized that the primary source of acrylamide exposure is nutrition, and more research should be conducted to monitor acrylamide exposure based on nutrition and associated health risks [21,22]. In this context, many studies have been conducted to examine the daily acrylamide exposure level and health risks by considering the nutritional habits of individuals living in different geographical locations [23,24,25,26,27,28,29]. However, to date, there has been no study conducted in Türkiye concerning this topic. The consequent lack of data prevents the necessary development of strategies required to reduce acrylamide exposure and health risks based on nutrition. The aim of this study is to determine the daily total acrylamide exposure level resulting from the consumption of some functional and traditional foods known to be frequently consumed in daily life by people living in Türkiye and to evaluate the exposure level in terms of carcinogenic, non-carcinogenic and neurotoxic health risks.

## 2. Materials and Methods

### 2.1. Samples

In total, nine food groups that are frequently and highly consumed by Turkish society in daily life and are known to have high acrylamide levels were included in this study. All information on food groups and their acrylamide levels are shown in Table 1.

### 2.2. Food Consumption Data and Scenarios

The Türkiye Nutrition and Health Survey was conducted in 2019 throughout Türkiye under the leadership of the Ministry of Health of the Republic of Türkiye with the cooperation of Hacettepe University, Başkent University, and Hasan Kalyoncu University. A total of 12,986 people (5824 males and 7162 females) participated in the study. Demographic characteristics of all participants, such as gender, age, and educational status, as well as individuals’ eating habits, food consumption information, and daily physical activities were recorded. Individuals’ consumption information regarding different food groups was obtained through face-to-face interviews using a 24-h recall method. The type and amount of different food groups consumed by individuals were recorded in detail using the “Food and Nutrition Photo Catalog-Measures and Quantities” book [36].

In this study, information on the consumption of bread (180 g/day), French fries (93.6 g/day), brewed black tea (416 mL/day), and brewed Turkish coffee (26 mL/day) was obtained from the Türkiye Nutrition and Health Survey [36]. However, there is no defined data on the consumption of desserts, traditional foods, chips, simits and breakfast cereals by individuals living in Türkiye. Therefore, the consumption amounts of these foods were determined by evaluating the portion amount defined on the packaging of the relevant foods or the consumption trends of these foods. Accordingly, the amount of portion consumed were accepted as 130 g, 100 g, 25 g, 60 g and 40 g for desserts, traditional foods, chips, simits and breakfast cereals, respectively.

Two different scenarios (good and bad scenarios) of consumption of these foods by the Turkish society were determined. In the good scenario, the consumption amounts of bread, brewed black tea and brewed Turkish coffee were not changed, while it was assumed that French fries, desserts, traditional foods, chips, simits and breakfast cereals were consumed once a week. In the bad scenario, it was assumed that all food groups were consumed every day.

### 2.3. Health Risk Assessment

#### 2.3.1. Dietary Acrylamide Exposure

Daily dietary acrylamide exposure levels were calculated using the equation below.

(1) $$\mathrm{EDI}=\frac{F\;\times \;C\;}{\mathrm{bw}}$$

EDI refers to the estimated daily acrylamide exposure (μg/kg bw/day); F is the amount of specific food consumed (g/day), C is the acrylamide concentration in the specific food (μg/kg-mL), and bw is the body weight (70 kg).

#### 2.3.2. Non-Carcinogenic Risk Assessment

For describing the non-carcinogenic health risk, the target hazard quotient (THQ) is widely applied. The hazard index (HI) is the sum of the THQs calculated for each food type. While THQ and HI ≥ 1 indicates a potential health problem that is non-carcinogenic, THQ and H < 1 means there is no concern about health risk [37]. THQ and HI were calculated using the following equations, respectively.

(2) $$\mathrm{THQ}=\frac{\mathrm{EDI}}{\mathrm{RfD}}$$

(3) $$\mathrm{HI}\;=\;{\mathrm{THQ}}_{\mathrm{Food}\;\mathrm{group}\;1}\;+\;{\mathrm{THQ}}_{\mathrm{Food}\;\mathrm{group}\;2}\;+\;\ldots \ldots \ldots \ldots \ldots \ldots \ldots \;+\;{\mathrm{THQ}}_{\mathrm{Food}\;\mathrm{group}\;n}$$

EDI stands for the estimated daily acrylamide exposure (μg/kg bw/day), and RfD (oral reference doses) determined for acrylamide is 2 × 10^−3^ mg/kg bw/day [38].

#### 2.3.3. Carcinogenic Risk Assessment

The carcinogenic risk (CR) assesses the cancer risk associated with a population’s lifetime exposure to a carcinogenic compound [39]. CR < 1.0 × 10^−6^ is considered safe, 1.0 × 10^−6^ < CR < 1.0 × 10^−4^ is considered a potential risk, and 1.0 × 10^−4^ < LCR is considered a serious health risk. CR was calculated using the following equation.

(4) CR=EDI × OSF

EDI stands for the estimated daily exposure (mg/kg b_w_/day), and OSF (oral slope factor) determined for acrylamide is 5 × 10^−1^ mg/kg b_w_/day [38].

#### 2.3.4. Risk Assessment according to the Exposure Margin Approach

The neurotoxic health risks of acrylamide exposure were assessed using the margin of exposure (MOE) approach. MOE was calculated using the equation below.

(5) $$\mathrm{MOE}=\frac{{\mathrm{NOAEL}\;\mathrm{or}\;\mathrm{BMDL}}_{10}}{\mathrm{EDI}}$$

MOE_n_ and MOE_c_ are MOE neurotoxic and MOE carcinogenic, respectively. No Observed Adverse Effect Level (NOAEL) was determined for acrylamide as 0.2 mg/kg bw/day for the morphological changes in nerves. Benchmark dose lower confidence limit (BMDL)_10_ value was determined as 0.31 (0.18) mg/kg bw/day for the induction of mammary tumors in rats (Harderian gland tumors in mice), respectively [22]. The lower the MOE is the greater risk it poses to humans.

## 3. Results and Discussions

### 3.1. Dietary Acrylamide Exposure Level

The daily total acrylamide exposure level of all individuals aged 15 and older living in Türkiye was calculated as 58 and 196 µg/day for the good and bad scenarios, respectively. Daily exposure levels of individuals according to their body weights are shown in Table 2.

Dietary acrylamide exposure levels based on body weight of individuals are estimated as 0.85 and 2.81 µg/kg bw/day for the good and bad scenarios, respectively. The JECFA reported dietary acrylamide exposures for the general population and highly exposed consumers as 1 and 4 µg/kg bw/day, respectively [40]. Tardiff et al. reported the tolerable daily intake for acrylamide-induced neurotoxicity and cancer as 40 and 2.6 µg/kg bw/day, respectively [41]. Daily total dietary acrylamide exposure has been reported as 0.43 µg/kg bw/day in Poland (1–96 years) [23], 0.29 µg/kg bw/day in Canada [25], 0.32 µg/kg bw/day in China (18–45 years) [26], 0.15 µg/kg bw/day in Japan (15–59 years) [27], 0.36 µg/kg bw/day in the United States (>2 years) [42], and 0.08 µg/kg bw/day in Korea (20–64 years) [28]. In a recent study conducted in Malaysia, acrylamide exposure levels of adult individuals with average and high food consumption were calculated as 0.23 and 1.77 µg/kg bw/day, respectively [29]. In the present study, the daily acrylamide exposure level calculated for the bad scenario is higher compared to tolerable daily intake value determined for cancer by Tardiff et al. [41]. Acrylamide exposure levels calculated for the good and bad scenarios are also higher compared to previous studies.

There are three main reasons for the significant difference between the studies. The difference in the foods included in the studies is the main determining factor. Even if the foods are similar, the acrylamide levels detected in the same food types will be significantly affected by many factors such as raw material properties, differences in the production process and the way consumers prepare their food. The amount and frequency of food consumption also varies according to geography, culture and the nutritional habits of people are another reason. Indeed, many researchers have pointed out the difficulty of making comparisons between existing data due to differences in consumers’ nutritional preferences, food preparation procedures and methods used to estimate acrylamide exposure among scientific studies [24,43,44].

### 3.2. The Contribution Level of Food Groups to Dietary Acrylamide Exposure

**Figure 1:**
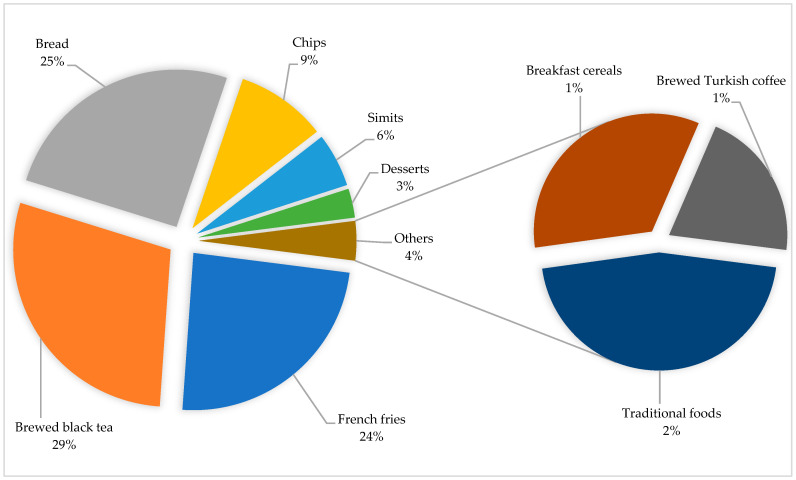
Contribution of foods (%) to daily dietary acrylamide exposure (good scenario).

**Figure 2:**
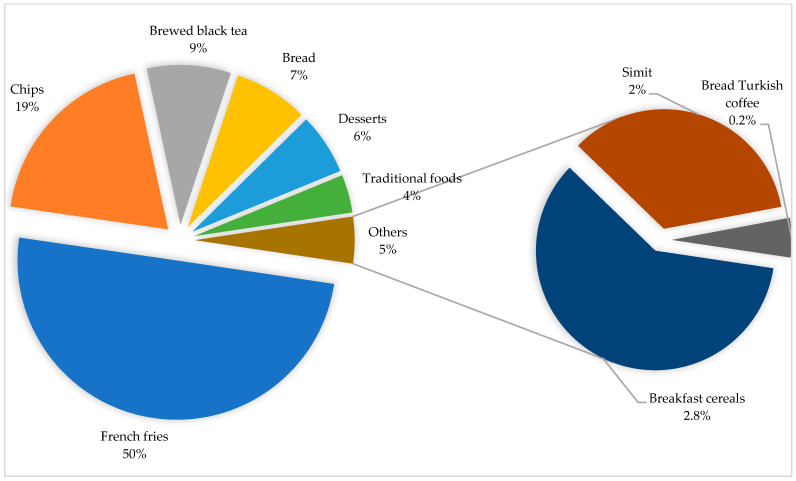
Contribution levels to daily dietary acrylamide exposure (bad scenario).

The contribution rates of the foods included in the study to estimate daily acrylamide exposure level of individuals aged 15 and older living in Türkiye, according to the good and bad scenarios, are shown in Figure 1 and Figure 2, respectively.

According to the good scenario, the daily acrylamide exposure level from each food consumption was calculated as 16.6 µg/day for brewed black tea, 14.7 µg/day for bread, 14 µg/day for fries, 5.4 µg/day for chips, 3.20 µg/day for bagels, 1.73 µg/day for desserts, 1.07 µg/day for traditional foods, 0.79 µg/day for breakfast cereals and 0.48 µg/day for brewed Turkish coffee. The contributions of brewed black tea, bread and French fries consumption to the total daily acrylamide exposure level are approximately 80%.

According to the bad scenario, the daily acrylamide exposure level arising from each food consumption was calculated as 97.7 µg/day for French fries, 37.8 µg/day for chips, 16.6 µg/day for brewed black tea, 14.7 µg/day for bread, 12.1 µg/day for desserts, 7.52 µg/day for traditional foods, 5.52 µg/day for breakfast cereals, 3.20 µg/day for bagels and 0.48 µg/day for brewed Turkish coffee. According to the bad scenario, the first five foods contributing the highest rate to daily acrylamide exposure level are French fries, chips, brewed black tea, bread and desserts with their total contribution levels greater than 90%. The contribution to daily acrylamide exposure of French fries consumption alone is 50%. In the good scenario, the consumption of foods with high acrylamide levels such as French fries, chips, and desserts is low, so their contribution to daily total acrylamide exposure is relatively lower than that of brewed black tea and bread. In the bad scenario, the consumption of these foods was increased, so their contribution to daily total acrylamide exposure has also changed significantly.

Tea is a popular beverage consumed worldwide. In this study, acrylamide exposure from brewed black tea consumption was 0.24 µg/kg bw/day, and the contribution of tea consumption to total daily acrylamide exposure was 29% (good scenario). Black tea contains relatively lower acrylamide levels than other foods [45,46]. On the other hand, in Türkiye, high consumption of black tea was a determinant for the high contribution rate to daily total acrylamide exposure. According to the Food and Agriculture Organization data in 2020, Türkiye ranks first in the world with 4 kg per capita tea consumption [47]. In a recent study, acrylamide exposure levels from brewed black tea consumption for the general population aged 15 years and over were determined to be in the range of 0.13–0.18 µg/kg bw/day [48]. In some previous studies conducted in countries with high tea consumption, such as Türkiye, the contribution of green tea consumption to total acrylamide exposure was reported as 21–23% [49,50]. On the other hand, in a study conducted in Korea, the contribution of tea consumption to daily acrylamide exposure level was reported as very low (2%) [51].

Bread is one of the basic food products frequently consumed in all meals from breakfast to dinner in many different geographies and cultures [52]. In this study, bread is in second place (25%) in terms of its contribution rate to the total daily acrylamide exposure (good scenario). Acrylamide exposure from bread consumption was calculated as 0.23 µg/kg bw/day. Approximately 120 million loaves of bread are produced per day in Türkiye [53] and Türkiye is considered one of the countries with the highest bread consumption in the world [54]. Although the acrylamide level of bread is low compared to other foods, its contribution to the total daily acrylamide exposure is high due to its high consumption. The European Commission has reported the benchmark level for acrylamide in bread as 50 µg/kg [55]. Due to its importance in nutrition, the acrylamide level of bread has been examined in many studies. Acrylamide levels in bread were reported as 57 µg/kg (Italy) [56], 135 µg/kg (Slovenia) [57] and 157 µg/kg (Iran) [58]. The same researchers reported the acrylamide exposure level from bread consumption as 0.19–0.26, 0.21 and 0.38 µg/kg bw/day, respectively. Mojska et al. reported that the highest contribution to the daily dietary acrylamide intake in adults was from bread consumption (49%) [23]. In a comprehensive study conducted in 27 centers in Europe, bread consumption was reported as the highest level of total daily acrylamide exposure (33%) [44]. Hirvonen et al. and McCullough et al. stated that bread consumption was the third highest contributor to daily acrylamide exposure (14 and 10%, respectively) [13,59]. Hidayah et al. reported that bread consumption contributed to daily acrylamide exposure as a very low value of 1% [29].

French fries are a popular fast-food product prepared by frying sliced potatoes in oil, served alone or with other dishes, and their consumption is increasing day by day [60]. Acrylamide exposure levels from French fries consumption are 0.20 and 1.40 µg/kg bw/day in the good and bad scenarios, respectively. The contribution of French fries consumption to daily acrylamide exposure level is approximately 24% (good scenario). The main reason for this situation is the high acrylamide level of French fries. The European Commission has declared the benchmark level for acrylamide in French fries (ready-to-eat) as 500 µg/kg [55]. High acrylamide levels in French fries have been reported in many studies in the literature [61,62,63]. The exposure level of acrylamide from the consumption of French fries has been reported as 0.20 µg/kg bw/day in France [24], 0.08 µg/kg bw/day in Spain [64], 0.66 µg/kg bw/day in Ethiopia [63], and 0.44 µg/kg bw/day in Romania [65]. Sirot et al. (France), Normandin et al. (Canada), and McCullough et al. (United States) reported that the highest contribution to total daily acrylamide exposure came from French fries, with 45, 50, and 23%, respectively [24,25,59]. The contribution of French fries consumption to daily acrylamide exposure was reported in previous studies as 14.6% (second highest contribution) [13], 13% (third highest contribution) [23], and 6.8% [29].

Chips are popular snacks produced with various spices and other additives for flavoring various grains and vegetables, especially corn and potato [66]. In this study, acrylamide exposure levels resulting from chip consumption were 0.08 and 0.54 µg/kg bw/day for the good and bad scenarios, respectively, and the contribution of chip consumption to the total daily acrylamide exposure was calculated as 9% (good scenario). Although the daily recommended portion size of chips (25 g) is lower than the consumption amounts of other foods, chips, like French fries, are among the foods that contain high levels of acrylamide. This situation explains the high contribution rate to the total daily acrylamide exposure level. The European Commission reported the benchmark level for acrylamide in potato chips as 750 µg/kg [55]. Hariri et al. reported the average acrylamide level in corn chips as 1574 µg/kg [67], Esposito et. al, and Sharafi et al. reported that potato chips contained acrylamide levels higher than 1500 µg/kg [68,69]. Different studies reported the acrylamide exposure levels resulting from potato chip consumption as 0.01 µg/kg bw/day [24] and 0.27 µg/kg bw/day [65]. The contribution of potato chip and corn chip consumption to the total daily acrylamide exposure level was reported as 10 and 5%, respectively [25]. Sirot et al. reported that the contribution of potato chips to the total daily acrylamide exposure level was as low as 2.4% [24]. Kawahara et al. and Hidayah et al. reported that the highest contribution to the total daily acrylamide exposure came from potato chips as 22% and 43.1%, respectively [27,29].

Simit is a product prepared according to the traditional production technique of a mixture of wheat flour, fresh yeast, sourdough, water and salt and baked in stone ovens. Simit is a food identified with Turkish society and is a geographical indication registered bakery product mostly consumed alone or as an addition to breakfast [35]. The contribution rate of simit consumption (0.05 µg/kg bw/day), which is consumed almost every day in Turkish society, to the total daily exposure is approximately 6% according to the good scenario. No research was found to compare the values obtained for simit in this study. Acrylamide levels in various bakery products have been reported as <30–640 µg/kg [70], 292–362 µg/kg [56], 199 µg/kg [58] and LOQ-47 µg/kg [71]. Svensson et al. reported acrylamide exposure levels from consumption of various bakery products (cookies/biscuits/wafers) [70], Sirot et al. in croissant-like pastries [24] and Cieslik et al. [71] reported in bakery products (kukiełka Lisiecka, obwarzanek, bagels and pretzels) as 0.02, 0.003 and 0.015 µg/kg bw/day, respectively, and the contribution of these levels to the total daily acrylamide exposure level was 5, 1.3 and 7.5%, respectively. Nematollahi et al. reported acrylamide exposure from consumption of bakery products as 0.25 µg/kg bw/day (17.7 µg/day) in individuals aged 18–60 living in Iran [58]. Keramat et al., Andačić et al. and Cieslik et al. estimated the contribution of bakery products to total daily acrylamide exposure as 20, 37.2 and 28% respectively [43,71,72]. However, acrylamide exposure from bread consumption was also added to these rates in two studies [43,72]. Burley et al. reported that bakery products were the second highest contributor to the total dietary acrylamide intake of women living in the UK as 17% [73].

The acrylamide exposure levels resulting from the consumption of sweet and traditional foods are 0.03 and 0.02 µg/kg bw/day, respectively, and the contribution of both foods to the daily total acrylamide exposure level is determined as 3 and 2%, respectively (good scenario). The sweet and traditional foods included in this study are registered with geographical indications in Türkiye and mostly consumed in only one meal, especially in restaurants. The consumption frequency of these foods is lower than other foods such as bread and tea. In addition, the acrylamide levels corresponding to one portion of these foods are also low compared to other foods included in the study. When both the consumption amount and acrylamide levels are considered, the contribution rates of these foods to the daily total acrylamide level are relatively low. Since the sweets and traditional foods included in this study are specific to Türkiye, no research could be found to directly compare the values obtained. Acrylamide levels have been reported as 26 µg/kg for cakes and other sweetened pastries [24], LOQ—3755 µg/kg for various traditional foods from Colombia [74] and 367 µg/kg for confectionery products [58]. Sirot et al. reported the acrylamide exposure level from consumption of cakes and other sweetened pastries as 0.013 µg/kg bw/day, which contributed 3% to the total daily acrylamide exposure level [24]. Cieslik et al. reported the acrylamide exposure level from consumption of cheesecakes as 0.03 µg/kg bw/day [71]. In a study (Korea) determining the acrylamide exposure levels resulting from the consumption of many processed foods frequently consumed in daily life, the contribution of sweet consumption to total daily acrylamide exposure was reported as 15.1%, and it was reported that this was the second highest contribution [51].

Breakfast cereals are products produced by enriching basic cereals with various nutrients and their consumption trend has increased especially in recent years [75]. The contribution of acrylamide exposure level (0.01–0.08 µg/kg bw/day) resulting from breakfast cereal consumption to daily acrylamide exposure level is 1–2.8%. Breakfast cereals are relatively new products for Turkish society. Therefore, due to both low consumption amount and acrylamide level, the contribution level of breakfast cereals to daily total acrylamide exposure has been limited. Acrylamide levels in breakfast cereals have been reported to range between 41–49 µg/kg in different studies [76,77]. The European Commission has declared the benchmark level for acrylamide in breakfast cereals as 300 µg/kg [55]. Acrylamide exposure levels from breakfast cereal consumption have been reported as 0.04 µg/kg bw/day in Spain [78], 0.001 µg/kg bw/day in France [24] and 0.07 µg/kg bw/day in Lebanon [76]. The contribution of breakfast cereals to dietary acrylamide exposure has been reported to range widely, varying between 0.2–8% in some studies [24,25,44,70].

Coffee is a beverage consumed by a large number of people worldwide. In this study, the acrylamide exposure level resulting from Turkish coffee consumption was 0.01 µg/kg bw/day, contributing approximately 1% to the total daily exposure level. Acrylamide exposure levels resulting from coffee consumption have been reported as 0.17 µg/kg bw/day [70], 0.08 µg/kg bw/day [23], 0.12 µg/kg bw/day [24]. The acrylamide exposure level resulting from the consumption of Lebanese traditional coffee has been reported as a very high value of 10.9 µg/kg bw/day [79]. Many studies have reported that coffee consumption contributes significantly to dietary acrylamide exposure by 39% [70], 40% [13], 27% [23], 28% [24], 24% [44], 15% [59] and 27% [51]. This significant difference in the results can be explained by two reasons. First, since Türkiye is a major tea producer, tea consumption as a hot beverage (mean: 416 mL/day) is quite common among the Turkish population. Therefore, coffee consumption (mean: 26 mL/day) is quite low. Second, considering the variety of coffees, consume of Turkish coffee, another geographically registered beverage, is quite common among the Turkish population. Turkish coffee is consumed in traditional cups, and these cups have an average volume of 70–80 mL. Considering that instant coffees and ready-to-drink coffees are consumed in a cup or paper cup of at least 200 mL, thus, the small volume of the cup preferred for Turkish coffee limits acrylamide exposure.

### 3.3. Health Risk Assessment

#### 3.3.1. Non-Carcinogenic Assessment

**Figure 3:**
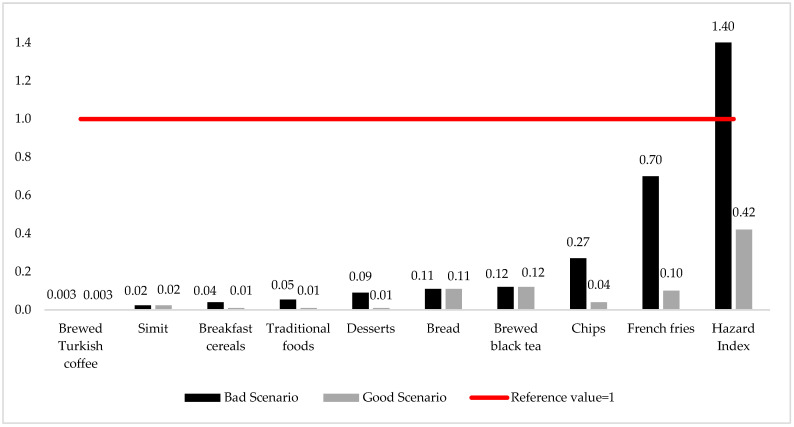
THQ values of foods according to dietary daily acrylamide exposure levels.

THQ and HI values were calculated according to the acrylamide exposure levels resulting from the consumption of each food and all foods for all individuals aged 15 and older living in Türkiye (Figure 3).

According to Figure 3, when both the good and bad scenarios are considered, the THQ value of each food is lower than the reference value of 1. Therefore, it can be said that the consumption of each food alone is safe in terms of non-carcinogenic health risks. In the good scenario, the top three foods with the highest THQ values are brewed black tea, bread and French fries, respectively, while in the bad scenario, they are French fries, chips and brewed black tea. According to the bad scenario, the THQ value calculated based on only the consumption of French fries is remarkable because it is close to 1. The main reason for this is that the acrylamide level of French fries is quite high compared to other foods. Therefore, a low potential health risk can be mentioned for individuals who consume French fries in large quantities.

Since individuals consume not only one food but many foods together in their daily lives, the cumulative health risks of acrylamide exposure from all foods were also examined in this study. The hazard index levels were determined as 0.42 and 1.40 for the good and bad scenarios, respectively. Therefore, the good scenario is reliable in terms of non-carcinogenic health risks. The bad scenario carries potential health risks. In the bad scenario, the daily consumption of foods with high acrylamide levels, especially French fries and chips, caused both the THQ values of the foods and the HI (total THQ) values to increase.

In various studies, THQ values for acrylamide exposure from consumption of brewed black tea, bread, potato chips and corn chips/breakfast cereals were reported as 0.08, 0.06, 1 and 0.06/0.04, respectively [30,48,69,80]. Hazard index values lower than 1 calculated according to daily acrylamide exposure level for individuals with normal and high consumption levels and explained that the non-carcinogenic risk was reported as negligible in previous studies [58,81].

#### 3.3.2. Carcinogenic and Neurotoxic Health Risk Assessment

Acrylamide exposure levels of the Turkish society resulting from the consumption of nine different foods were examined in terms of carcinogenic health risks and the values obtained are shown in Table 3.

According to the good scenario, CR levels of acrylamide exposure levels resulting from the consumption of brewed Turkish coffee, simit, breakfast cereals, traditional food, desserts, chips and French fries are in the range of 1 × 10^−4^–1 × 10^−6^, and consumption of these foods indicates a possible health risk in terms of carcinogenicity. The CR levels of acrylamide exposure levels resulting from the consumption of bread, brewed black tea and all foods indicate the existence of a serious health risk.

According to the bad scenario, CR levels of acrylamide exposure levels resulting from consumption of brewed Turkish coffee, simit, breakfast cereals, traditional food and desserts indicate a potential and significant carcinogenic health risk. Since CR levels of acrylamide exposure levels resulting from consumption of bread, brewed black tea, chips, French fries and consumption of all foods are greater than the critical reference value of 1 × 10^−4^, and indicate a serious danger in terms of carcinogenic health risks.

When the CR values of both scenarios are taken into account, it is clear that there is a carcinogenic health risk. This situation is directly related to the acrylamide level of the food and the amount of food consumed. Therefore, individuals need to consume less foods containing high acrylamide levels in their daily lives or reduce their frequency of consumption.

In different studies, CR values for acrylamide exposure from consumption of brewed black tea, bread, potato chips and corn chips/breakfast cereals were reported as 6.46 × 10^−5^–9.12 × 10^−5^, 1.83 × 10^−5^–11.1 × 10^−5^, 6.30 × 10^−5^ and 5.66 × 10^−5^/3.95 × 10^−5^, respectively [30,48,69,80]. Nematollahi et al. reported CR values for adults with normal and high levels of consumption, based on daily dietary acrylamide exposure levels of individuals living in Iran, as 1.89 × 10^−4^ and 4.17 × 10^−4^, respectively [58]. In a recent study, CR values were reported in the range of 2.05 × 10^−4^–2.78 × 10^−4^ according to acrylamide exposure levels resulting from consumption of bread, French fries and coffee in pregnant women of different ages and trimesters [81].

**Figure 4:**
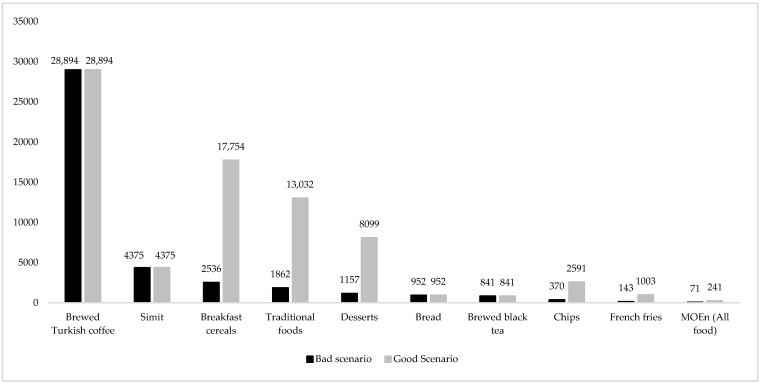
MOE_n_ values of foods based on daily total dietary acrylamide exposure levels.

In this study, the values obtained for acrylamide exposure were evaluated in terms of neurotoxicity with a different health risk assessment method, MOE_n_ (Figure 4).

According to the good and bad scenarios, the MOE_n_ values of the foods are in the range of 841–28,994 and 143–28,994, respectively, and the MOE_n_ values for acrylamide exposure resulting from the consumption of all foods are 241 and 71, respectively. JECFA has defined the MOE_n_ values for acrylamide based on nutrition as 200 for individuals with average consumption [22]. Therefore, when the MOE_n_ value of each food is examined, it can be said that only the acrylamide exposure level resulting from the consumption of French fries defined in the bad scenario has neurotoxic health risks. The MOE_n_ values calculated according to the total daily acrylamide exposure levels indicate that negative neurological effects are unlikely for the good scenario, whereas there is a neurotoxic health risk for the bad scenario. However, it should still be noted that the value calculated for the good scenario (241) is close to the critical limit. Therefore, even a partial increase in the consumption of foods in the good scenario may lead to the manifestation of neurological health risks.

In similar studies, MOE_n_ values for acrylamide exposure resulting from consumption of different foods were reported as 628 [26], 18–535 [79], 1818–3509 [82], 286 [76], 303 [63], 156–2534 [48] and 556–1667 [30].

**Figure 5:**
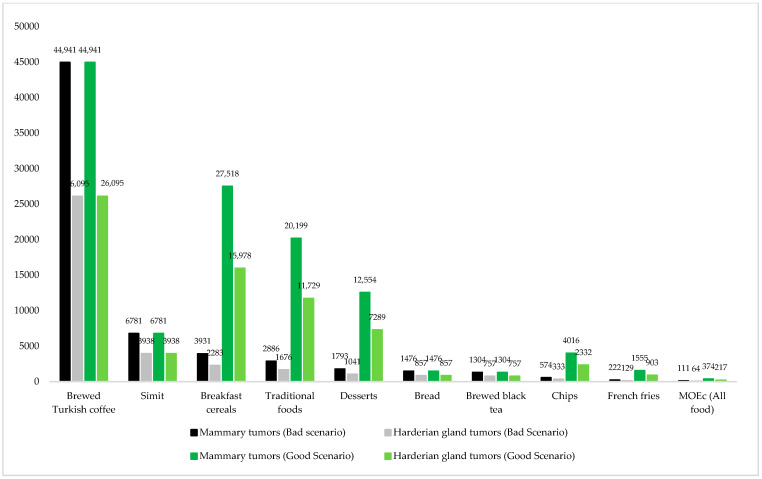
MOE_c_ values of foods based on daily total dietary acrylamide exposure levels.

The values obtained for dietary acrylamide exposure were also evaluated with MOE_c_ for carcinogenicity (Figure 5).

According to the good and bad scenarios, the MOE_c_ values of the foods ranged from 1304 (757)–44,941 (26,095) and 222 (129)–44,941 (26,095), respectively. The limit values for MOE_c_ (mammary tumors (Harderian gland tumors)) are 310 (180) [22]. Accordingly, the consumption of all foods except French fries was found to be safe in terms of carcinogenicity. According to the total daily acrylamide exposure levels, the MOE_c_ values of the bad scenario (111 (64)) were lower than the reference values, which means that there are carcinogenic health risks. The good scenario (all food) was evaluated as safe in terms of carcinogenicity.

MOE_c_ values for daily acrylamide exposure was 721 (418) for the consumption of 212 different foods [24], 109–1069 (63–621) for the consumption of deep-fried French fries, oven-baked French fries, potato chips, corn chips, popcorn, pretzels, roasted almonds, crackers, cookies, chocolate chips cookies, breakfast cereals, bread [25], 973 (565) for consumption of black olives and brewed coffee, cereals, legumes and nuts, potatoes, meat, eggs, aquatic foods, milk, vegetables, fruits, sugars, water and beverages, and alcoholic beverages [26] 28–829 (17–481) for consumption of caffeinated beverages [79], 4042 (2347) for consumption of potato crisps, crisps (except potato crisps), biscuits, French fries, chocolate products, cocoa products, breakfast cereals, tea products, nut and nut products, dried and roasted seaweed, coffee substitutes, bread, cakes, juices, and some other traditional products [28], 67–1550 (39–900) for consumption of cereal-based foods as [76] and 470 (272) for consumption of coffee, potato chips and French fries [63]. However, it should be noted that the MOE_c_ values of the good scenario are close to the critical limit and that, just like in MOE_n_, an increase in the consumption of the relevant foods may create concerns in terms of carcinogenic health risks.

The differences between the THQ, HI, CR and MOE_n_ and MOE_c_ values calculated for dietary acrylamide exposure in this study and other studies are most likely due to differences in acrylamide levels of the foods included and the changes in the consumption of these foods according to societies.

### 3.4. Limitations

This study included chips, breakfast cereals and breads of different brands sold in Türkiye. The study also included French fries, tea and Turkish coffee prepared considering the nutritional habits of the Turkish society, and some traditional foods ready for consumption. Many factors such as raw material properties, production methods, cooking techniques and storage properties of these foods, which are sold packaged or consumed after a preparation stage, are different from each other. Therefore, the sensory and nutritional properties and acrylamide levels of the foods included in this study may differ from similar foods. In addition, the nutritional habits of individuals may vary according to many factors such as age, gender, geography and culture. It is worth noting that both situations may directly affect individuals’ dietary acrylamide exposure and associated health risks. It is clear that not all food products produced and consumed in Türkiye that may contain acrylamide cannot be evaluated in such a study. It should also be remembered that individuals may consume foods other than the foods included in this study that may contain acrylamide. Therefore, it should be taken into account that individuals’ acrylamide exposure may be higher than the values reached in this study.

## 4. Conclusions

Acrylamide has been recognized as a contaminant in foods for over 20 years, and is still being studied in the scientific world today due to its potential health risks for humans. In this study, the health risks of people living in Türkiye were assessed according to two different scenarios for dietary acrylamide exposure. Acrylamide exposure levels resulting from the consumption of each of the studied nine foods and all foods together are high compared to other studies in the literature. Accordingly, the HI and MOE_n_ and MOE_c_ values of each food and all foods in the good scenario were evaluated as safe in terms of health risks. The HI, MOE_n_ and MOE_c_ values of the bad scenario (for all foods) indicate the presence of significant health risks. The CR values of both the good and bad scenarios were evaluated as risky. All this information clearly demonstrates that there are potential health concerns regarding dietary acrylamide exposure in Turkish society. To reduce acrylamide exposure through dietary intake, individuals should focus on a balanced diet, especially one that incorporates fruits and vegetables. Moreover, it is advisable to minimize the quantity and frequency of consuming foods containing high acrylamide levels. In addition, consumers and the food industry should implement practices to reduce acrylamide levels in food preparation stages (steaming, boiling, etc.) and in production stages. This study is the first to evaluate acrylamide exposure and health risks based on nutrition in Türkiye. In this context, it is very important for public authorities and universities to periodically conduct similar studies to increase the number and variety of foods for future strategies regarding the prevention of acrylamide exposure. In this context, studies aimed at reducing acrylamide levels in foods should be prioritized. Consumers can then be taught practical applications to reduce acrylamide levels in foods. Finally, consumers should be informed to consume less foods with high acrylamide content.

## Figures and Tables

**Table 1:** Food groups included in the study and acrylamide levels.

Food Groups	Acrylamide Level (Mean ± SD)	Acrylamide Level (Min.–Max.)	Types of Food	References
Chips	1513 ± 1876 µg/kg	256–7423 µg/kg	Potato chips (*n* = 11); Corn chips (*n* = 19)	[30]
French fries	1039 ± 76 µg/kg	966–1144 µg/kg	The acrylamide level of French fries was determined by taking the average of all the data obtained by frying 4 different types of oil (sunflower oil, olive oil, corn oil, and hazelnut oil) for 8 consecutive times.	[31]
Breakfast cereals	138 ± 14 µg/kg	41–362 µg/kg	Breakfast cereals (*n* = 20)	[30]
Breads	81.7 ± 14 µg/kg	61–130 µg/kg	Multi-grain bread (*n* = 12); Whole-meal bread (*n* = 10); Whole wheat bread (*n* = 12); Rye bread (*n* = 8); White bread (*n* = 10)	[32]
Brewed black tea	40 ± 14 µg/L	25–74 µg/L	Black tea (*n* = 20)	[33]
Brewed Turkish coffee	1.3 ± 0.7 µg/1 cup (70 mL)	0.8–1.2 µg/1 cup (70 mL)	Turkish coffee (*n* = 4)	[34]
Desserts	12.1 ± 11 µg/1 portion (130 g)	1.2–37 µg/1 portion (130 g)	Baklava (pistachio) (*n* = 5); Künefe (*n* = 3); Kaymaklı ekmek kadayıfı (crumpets in thick syrup) (*n* = 3); Halka dessert (*n* = 3); Lokma dessert (*n* = 3); Tulumba dessert (*n* = 3); Laz böreği (*n* = 2)	[35]
Traditional foods	7.50 ± 5.6 µg/1 portion (100 g)	3–13 µg/1 portion (100 g)	Adana kebab (*n* = 3); Lahmacun (*n* = 3); Akhisar meat patty (*n* = 2); İnegöl meat patty (*n* = 2); Akçaabat meat patty (*n* = 3); Kavurma (*n* = 2); Stuffed meatball (*n* = 2)	[35]
Simits	3.20 ± 2.8 µg/1 portion (60 g)	0.75–6.35 µg/1 portion (60 g)	Rize simit (*n* = 3); Samsun simit (*n* = 3); Ankara simit (*n* = 3)	[35]

**Table 2:** Acrylamide exposure from consumption of different foods according to body weight.

Scenarios	Desserts	Traditional Foods	Simit	French Fries	Bread	Brewed Tea	Brewed Turkish Coffee	Chips	Breakfast Cereals	Total Acrylamide Exposure (µg/kg bw/day)
Good scenario	0.03	0.02	0.05	0.20	0.21	0.24	0.01	0.08	0.01	0.85
Bad scenario	0.17	0.11	0.05	1.40	0.21	0.24	0.01	0.54	0.08	2.81

**Table 3:** CR levels of foods according to daily acrylamide exposure levels based on diet.

Food Groups	Good Scenario	Bad Scenario
Brewed Turkish coffee	3.45 × 10^−6^	3.45 × 10^−6^
Simit	2.29 × 10^−5^	2.2 × 10^−5^
Breakfast cereals	5.63 × 10^−6^	3.94 × 10^−5^
Traditional foods	7.67 × 10^−6^	5.37 × 10^−5^
Desserts	1.23 × 10^−5^	8.64 × 10^−5^
Bread	1.05 × 10^−4^	1.05 × 10^−4^
Brewed black tea	1.19 × 10^−4^	1.19 × 10^−4^
Chips	3.86 × 10^−5^	2.70 × 10^−4^
French fries	9.97 × 10^−5^	6.98 × 10^−4^
Daily total CR	4.14 × 10^−4^	1.40 × 10^−3^

## Data Availability

The data presented in this study are available on request from the corresponding author.
